# Case report: Hypoadrenocorticism crisis complicated by non-cardiogenic pulmonary edema in a dog

**DOI:** 10.3389/fvets.2022.1015739

**Published:** 2022-11-10

**Authors:** Mathieu V. Paulin, Elisabeth C. Snead

**Affiliations:** Department of Small Animal Clinical Sciences, Western College of Veterinary Medicine, University of Saskatchewan, Saskatoon, SK, Canada

**Keywords:** acute lung injury, acute respiratory distress syndrome (ARDS), neurogenic pulmonary edema, adrenal insufficiency, case report, addison crisis

## Abstract

A 6-year-old castrated male Labradoodle was referred in uncompensated hypovolemic shock, with a 72**-**h history of lethargy, vomiting and diarrhea that had acutely worsened with subsequent development of profuse hemorrhagic diarrhea in the last 24 h after a visit to the groomer. In most respects this case was classic for a patient with a primary hypoadrenocortical crisis. After initial attempts to address hypovolemia and refractory hypotension, no clinical improvement was seen, and the respiratory rate had increased acutely to 80 bpm with crackles detected on thoracic auscultation and serosanguineous fluid began draining from the nose and mouth. An arterial blood gas sample while breathing room air revealed moderate hypoxemia (PaO_2_ 59.9: RI 95–100 mmHg), an elevated alveolar-arterial (A-a) gradient at 54.7 (RI < 15 mmHg) and a PaO_2_:FiO_2_ ratio of 285 mmHg. Thoracic radiographs revealed severe bilateral alveolar lung pattern largely limited to the perihilar and caudodorsal lung fields. The radiographic findings, along with signs of ongoing hypovolemia, the lack of evidence of typical long-standing acquired cardiac disease, and the rapid resolution of the pulmonary edema without the need for diuretics or long-term cardiac medications supported non-cardiogenic pulmonary edema. The proposed cause of the non-cardiogenic pulmonary edema was speculated to be neurogenically mediated. Oxygen supplementation along with mineralocorticoid and glucocorticoid replacement therapy was sufficient for the management of the non-cardiogenic pulmonary edema in this case.

## Introduction

Hypoadrenocorticism is an uncommon condition in dogs, and is most often caused by immune-mediated destruction of the adrenal glands resulting in decreased mineralocorticoid and glucocorticoid production (typical primary hypoadrenocorticism) (1). Definitive diagnosis requires adrenocorticotropic hormone (ACTH) stimulation testing to demonstrate low basal and post-ACTH cortisol levels. The prognosis for hypoadrenocorticism is excellent with appropriate mineralocorticoid and glucocorticoid supplementation (1, 2). An acute hypoadrenocortical crisis in a dog represents a true medical emergency due to the resulting severe hypovolemia, dehydration, hypotension, electrolyte derangements, and acid-base abnormalities. The objective of this case report is to describe a hypoadrenocorticism crisis in a dog, whose medical management was complicated by refractory severe hypotension and subsequent development of non-cardiogenic pulmonary edema leading to arterial hypoxemia. This case report was written in agreement with the most updated version of the CARE guidelines (3).

## Case presentation

A 6-year-old, 25 kg, castrated male Labradoodle initially presented to the referring veterinarian (RDVM) with a 72-h history of lethargy, vomiting and diarrhea, that had acutely worsened with subsequent development of profuse hemorrhagic diarrhea in the last 24 h after a visit to the groomer. At the RDVM, abnormal physical examination findings included lateral recumbency with generalized muscle weakness, decreased mentation, poor peripheral pulses, capillary refill time (CRT) > 3 s, a 3-s skin tent, bradycardia (60 bpm), a low normal temperature (37.5°C) and abdominal pain. Intravenous (IV) crystalloid fluid therapy (Isolyte, B. Braun) was initiated at a rate of 78 mL/h. Blood work prior to initiation of any therapy revealed mild hemoconcentration [hematocrit (HCT) 57.4; RI 37–55%], lack of a stress lymphopenia (3.6; RI 1.2–4.5 × 10^9^/L), moderate azotemia (urea 34.3; RI 2.5–8.9 mmol/L, creatinine 584; RI 27–124 umol/L), mild hyponatremia (135; RI 138–160 mmol/L), along with severe hyperkalemia (>8.5; RI 3.7–5.8 mmol/L) with a sodium to potassium ratio that was low at 15, mild hypercalcemia (3.11; RI 2.15–2.95 mmol/L), hyperphosphatemia (3.68; RI 0.94–2.13 mmol/L), mild hypoglycemia (3.1; RI 3.3–6.1 mmol/L), borderline hypoalbuminemia (25; RI 25–44 g/L) with a normal serum total protein concentration (78; RI 54–82 g/L), and a mild elevation in alanine transferase (161; RI 10–118 U/L). Hypoadrenocorticism was suspected by the RDVM, and the fluid rate was increased to 156 mL/h with 2.5% dextrose supplementation. Dexamethasone (0.25 mg/kg IV) and hydromorphone [0.05 mg/kg intramuscular (IM)] were administered, and an ACTH stimulation test (250 ug IV) was performed. There was no response to treatment and the dog became more depressed and remained in lateral recumbency. A repeat serum biochemistry profile showed persistent marked hyperkalemia (8.2; RI 3.7–5.8 mmol/L) with worsening hyponatremia (127; RI 138–160 mmol/L), mild hypoalbuminemia (20, RI 25–44 g/L) with borderline hypoproteinemia (56; RI 54–82 g/L). Fludrocortisone [0.25 mg *per os* (PO)] was administered, but again due to lack of improvement, the patient was referred for further care. Prior to referral, a total volume of 546 mLs (or 21 mL/kg) IV crystalloid fluids had been administered.

On presentation (*t* = 0h) to the Emergency Service of the Western College of Veterinary Medicine, the dog was in lateral recumbency and non-responsive to external stimuli. Mucus membranes were pale, tacky with a prolonged CRT (>3 s). Hypothermia (36.2**°**C) and cold extremities, bradycardia (68 bpm), tachypnea (40 bpm), poor peripheral pulses, and melena on rectal examination were noted. Oscillometric blood pressure measurement revealed hypotension [systolic (SAP), diastolic (DAP), and mean (MAP) arterial pressure of 85/36 (54) mmHg, respectively]. No heart murmur was detected on auscultation. ECG findings revealed an inconsistent presence of P waves and a widening of the QRS complex suspicious for atrial standstill. Point of care blood work revealed a normal HCT (42%), low normal TP (5.5; 5.5–7.5 g/dL), hyperglycemia (12.3; RI 3.5–11.5 mmol/L), and a marked elevation in blood urea nitrogen (50–80 mmol/L—Azostix^®^ Reagent). Uncompensated hypovolemic shock was suspected and 20 mL/kg IV of 10% pentastarch was administered (4), along with four 10 mL/kg boli of 0.9% NaCl each given over 15 min with repeat assessment of CRT, blood pressure and peripheral pulse quality (5). The synthetic colloid was administered to help limit the volume of IV crystalloid fluids required in order to avoid potential exacerbation of the hyponatremia and a sudden decrease in the serum osmotic pressure.

**Figure 1:**
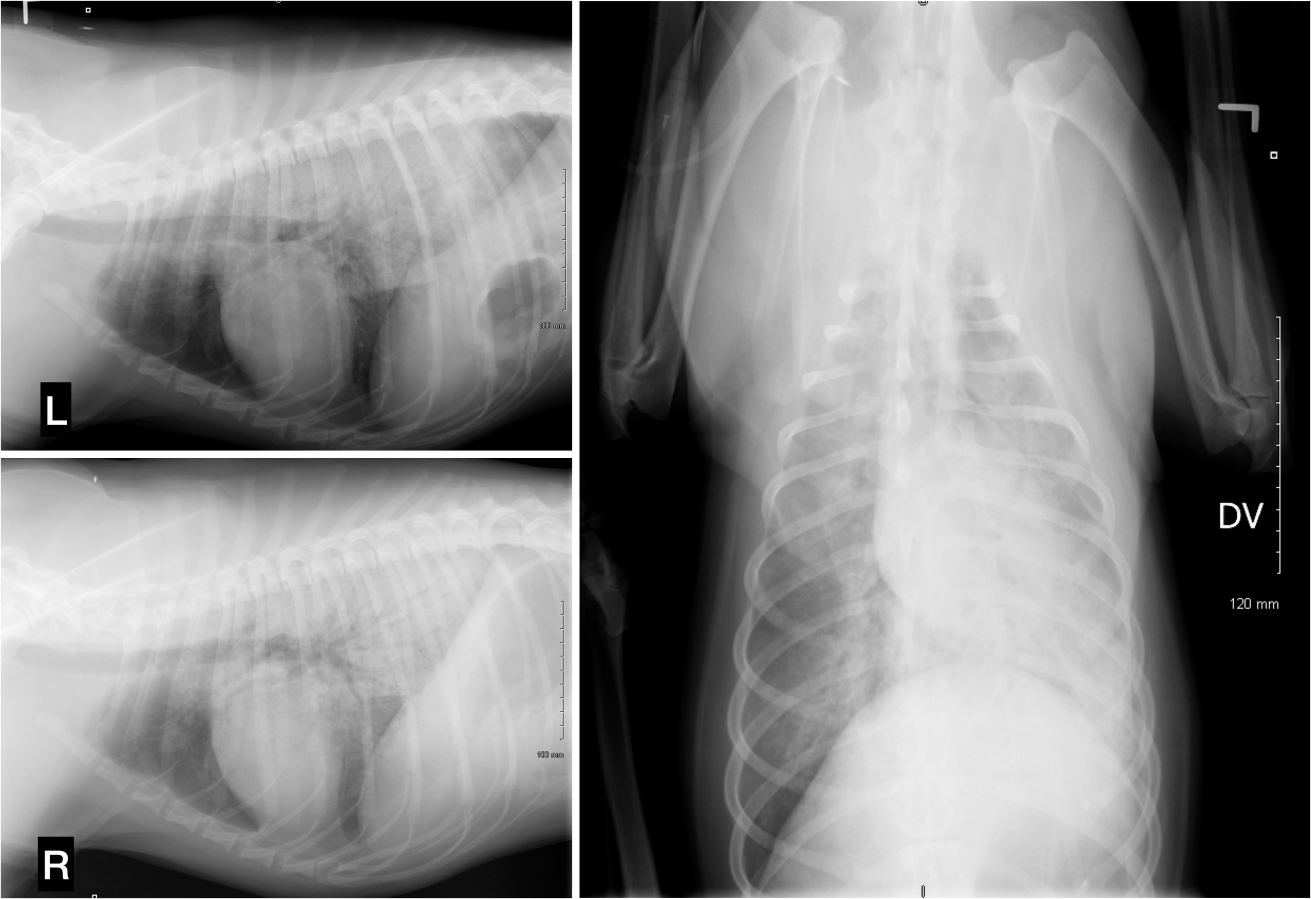
Thoracic radiographs, 3 projections (left lateral, right lateral, and dorsoventral). A severe alveolar lung pattern is present in the perihilar region and caudodorsal lungs, mainly distributed on the right side. There is a moderate diffusely distributed broncho-interstitial lung pattern in the left cranial and right cranial and right middle lung lobes. The cranial pulmonary vessels are very small and there is a narrowing of the caudal vena cava. The aorta cannot be identified due to superimposition. Trachea, cardiac silhouette, and diaphragm appear within normal limits.

At *t* = 2h, no clinical improvement was seen, and the respiratory rate had increased acutely to 80 bpm with crackles detected on thoracic auscultation and serosanguineous fluid began draining from the nose and mouth. The dog's head was kept in a dependent position to promote drainage. An arterial catheter was placed in the dorsal pedal artery. An arterial blood gas (ABG) sample at room air revealed moderate hypoxia (PaO_2_ 59.9: RI 95–100 mmHg), an elevated alveolar-arterial (A-a) gradient at 54.7 (RI < 15 mmHg), a moderate metabolic acidosis (pH 7.27, BE −11.9 mmol/L, HCO_3_-13.3 mmol/L) with compensatory respiratory alkalosis (PaCO_2_ 29.1, RI 35–40 mmHg). The calculated PaO_2_:FiO_2_ (fraction of inspired oxygen) ratio was 285 mm Hg (59.9:0.21). Marked hyperkalemia (7.8 mmol/L, RI 3.7–5.8 mmol/L), worsening hyponatremia (124 mmol/L, RI 138–160 mmol/L), normochloremia (106; RI 105–120 mmol/L), and normolactatemia (1.06, RI 0–2.0 mmol/L) were also noticed. Deoxycorticosterone pivalate (DOCP, 2.2 mg/kg IM), was administered to provide rapid onset mineralocorticoid supplementation within hours (2), and a bolus of 0.5 mL/kg of 50% dextrose diluted 1:4 with 1 unit of regular insulin subcutaneous (SC) was given for management of hyperkalemia. Oxygen supplementation was provided by bilateral nasal O_2_ lines (3 L/min). Respiratory rate and effort both decreased with oxygen supplementation. Thoracic radiographs revealed a severe alveolar lung pattern largely limited to the perihilar and caudodorsal lung fields, worse on the right than the left (Figure 1) along with microcardia, narrowed cranial pulmonary vessels and caudal vena cava suggesting ongoing hypovolemia. The distribution of the pulmonary edema was consistent with non-cardiogenic pulmonary edema, but cardiogenic pulmonary edema or atypical pneumonia could not be definitively ruled out. Hypotension persisted despite ongoing IV 0.9% NaCl fluid therapy at 130 mL/h with 2.5% dextrose supplementation and 5 mL/kg/h IV CRI of 10% pentastarch added to the fluid therapy. At that time, isosthenuria (urine specific gravity of 1.014) was noted on point of care urinalysis. Urine was collected by catheterization.

At *t* = 3h, early disseminated intravascular coagulation (DIC) was suspected based on moderate thrombocytopenia (67; RI 200–900 × 10^9^/L) and a mild elevation of prothrombin (PT) (18, RI 11–17 s) and partial thromboplastin times (PTT) (109, RI 72–102 s). Broad-spectrum antimicrobial therapy was initiated (ampicillin 22 mg/kg IV q6h, and enrofloxacin 10 mg/kg IV q24h) to cover any possible bacterial translocation from gastrointestinal ulceration due to suspected hypocortisolemia and ischemia related to shock, along with pantoprazole (1 mg/kg IV q24h). A dobutamine CRI at 5 ug/kg/min was initiated for inotropic support to help combat the refractory hypotension.

At *t* = 4h, hypotension persisted with minimal improvement of hyperkalemia (7.07; RI 3.7–5.8 mmol/L), and worsening of the metabolic acidosis (pH 7.0, BE −14.1 mmol/L). On continuous ECG monitoring, the previously reported changes (inconsistent P waves with widening of the QRS complex) had, however, resolved. The dobutamine IV CRI was increased to 15 ug/kg/min and phenylephrine IV CRI (0.25 ug/kg/min) was initiated, however, the dog's direct MAP remained < 60 mmHg. Pentastarch IV CRI was discontinued, a fresh frozen plasma transfusion was initiated (500 mL IV over 2 h), and a second dose of dexamethasone was given IV (0.25 mg/kg).

At *t* = 5h, arterial blood gas (with the patient off nasal oxygen for more than 5 min) revealed mild improvement in the degree of hypoxia (PaO_2_ = 78 mmHg, RI 95–100 mmHg) and mild hypercarbia (PaCO_2_ = 44 mmHg, RI 35–40 mmHg) with PaO_2_/FiO_2_ improved to 371 mmHg. Hyperkalemia had resolved (4.9 mmol/L) and the hyponatremia had improved (135 mmol/L) but the metabolic acidosis remained severe (pH 7.14, BE −14.9 mmol/L). The patient remained hypotensive (direct MAP < 60 mmHg), and hypothermia had worsened (34.7**°**C). Vasopressin IV CRI (1 microunit/kg/min) was initiated and within 20 min, direct arterial blood pressure improved with SAP, DAP, and MAP of 100/60 (82), respectively.

Between *t* = 5h and *t* = 10h, mean blood pressure steadily improved to 90 mmHg. The vasopressin IV CRI was discontinued at *t* = 12h, and dobutamine and phenylephrine IV CRIs were discontinued at *t* = 15h after presentation. Over the same time frame, tachypnea and hypothermia also resolved (36 bpm, 37.6**°**C, respectively). Oxygen supplementation was tapered down and discontinued at *t* = 37h. The patient's HCT and TP remained stable at 35% and 5.6 g/dL, respectively, and the automated platelet count increased steadily to 147 (RI 200–900 × 10^9^/L). Over the period of hospitalization, an indwelling urinary catheter was not placed, and the patient underwent intermittent urinary catheterization until able to urinate on his own once ambulatory. Neither anuria nor oligoanuria were noticed and urine output was more than 2 mL/kg/h based on quantification of urine output every 4–6 h while recumbent.

The results of the ACTH stimulation test confirmed hypocortisolism (pre- and post-cortisol <20 nmol/L). Dexamethasone (0.25 mg/kg IV q12h) was continued for three days following admission, then replaced with oral prednisone therapy (0.8 mg/kg PO q24h) and IV fluid therapy was slowly discontinued. The patient had markedly depressed mentation for the first 24 h but no other neurological signs (such as nystagmus, seizures) were noted. On day 5, the dog was discharged with oral amoxicillin, enrofloxacin and omeprazole for an additional 7 days, in addition to the daily prednisone therapy that was slowly weaned down over 7 days to a physiologic dose of 0.3 mg/kg PO q24h.

Over the course of the next month, the dog was examined several times. He returned to normal health and all abnormalities on blood work resolved. On day 32, the dog was started on fludrocortisone (0.25 mg PO q12h) to reduce costs associated with mineralocorticoid replacement therapy. Over more than 52 months of follow-up available, the dog has done well with no signs of recurrence of pulmonary edema or signs of underlying cardiac disease.

## Discussion

In most respects, this case was classic for a patient presenting with a primary hypoadrenocorticism crisis (6). However, the development of suspect non-cardiogenic pulmonary edema (NCPE) was an unusual feature and complicated the case management. In the literature, several mechanisms have been suggested for the development of NCPE, including injury to the alveolar-capillary epithelial membranes, a local increase in the pulmonary transcapillary hydrostatic pressure without an increase in left atrial pressure, or both. These mechanisms can lead to disruption of gap junctions between the cells resulting in exudation of protein-rich pulmonary edema (7–10).

Cardiogenic pulmonary edema (CPE) and NCPE are challenging to distinguish, given their similar clinical presentations (tachypnea, dyspnea, arterial hypoxia, cough, and expectoration of frothy edema fluid). However, differentiation is important due to implications for both treatment and prognosis. Radiographic findings of NCPE include a normal cardiac silhouette (normal or small vertebral heart score), normal to small vasculature with a patchy alveolar or interstitial pattern commonly noted in the caudodorsal lung fields (7). Hypoxia (PaO_2_ < 80 mmHg) and an elevated alveolar-arterial (A-a) gradient are expected on arterial blood gas analysis due to the resulting ventilation-perfusion mismatch (7). Echocardiography typically reveals normal sized cardiac chambers with normal cardiac contractility, in addition to a normal ECG (7). Primary hypoadrenocorticism has been reported as a rare cause of cardiac dysfunction in human patients, with reversible cardiac structural abnormalities following treatment, and systolic dysfunction has been seen following the initiation of mineralocorticoid replacement therapy (11–13). The latter was speculated to be secondary to chronic upregulation of renal adaptative mechanisms to limit water and sodium loss which, with subsequent introduction of mineralocorticoid supplementation, lead to excessive sodium and water retention and volume overload (12). Recently, three dogs were concurrently reported with hypoadrenocorticism and ventricular dilation with systolic dysfunction; two dogs presented with clinical signs consistent with biventricular congestive heart failure and a third dog presented with signs of acute hypoadrenocorticism without congestive heart failure (14). Stress cardiomyopathy (Takotsubo cardiomyopathy) is a form of reversible transient systolic dysfunction of the apical portion of the left ventricle triggered by severe emotional or physiological stress that typically resolves rapidly (15–17). The underlying pathophysiology is uncertain but may result from a transient and often undetected catecholamine overload. Takotsubo cardiomyopathy has been reported with human hypoadrenal crisis (15–19), with improvement following steroid treatment (18). CPE in a dog following initiation of therapy for concurrent hypoadrenocorticism and hypothyroidism has also been reported, and pulmonary edema resolved following treatment with pimobendan and furosemide (20). Regrettably, there was no cardiology service available to perform an echocardiogram in the reported dog. Measurement of proBNP or cardiac troponin enzymes prior to fluid therapy could have been useful for differentiating CPE from NCPE in this case but these were not measured for practical and financial reasons. In this dog, the radiographic distribution of the pulmonary edema along with signs of ongoing hypovolemia, the lack of evidence of typical long-standing acquired cardiac disease, and the rapid resolution of the pulmonary edema without the need for diuretics or long-term cardiac medications supported NCPE (7, 21). The owner declined recheck thoracic radiographs due cost constrain but it would have been pertinent to document the suspected fast resolution of the radiographic changes.

Most of the well-recognized causes of NCPE including head trauma, electric shock, smoke inhalation, near drowning, upper airway obstruction, were discounted (7, 22). The rapid resolution of the pulmonary edema led us to believe that the pulmonary edema was attributed to neurogenic pulmonary edema (NPE) rather than acute lung injury (ALI) or acute respiratory distress syndrome (ARDS) which are other subcategories of NCPE (7, 23). The NPE here was suspected to be secondary to central nervous system (CNS) injury due to hypoglycemia (24), hyponatremia (25, 26), or a combination of those.

Acute lung injury (ALI) and ARDS each represents part of a spectrum of a complex, multiphase clinical syndrome that leads to progressive respiratory failure. Both are characterized by rapid onset, diffuse, bilateral lung injury, severe hypoxemia, NCPE, low alveolar ventilation/perfusion ratios (VA/Q), and abnormal physiological shunting of oxygen (A-a gradient) with affected patients typically requiring prolonged mechanical ventilatory support (days to weeks). Both entities can result from severe systemic diseases [e.g., sepsis, systemic inflammatory response syndrome (SIRS), pancreatitis, and severe hyperuremia] and are differentiated by the degree of oxygen desaturation that occurs; patients with the less severe form (ALI) have a PaO_2_:FiO_2_ ratio ≤ 300 mmHg while with the more severe form (ARDS), they have a PaO_2_:FiO_2_ ratio of ≤ 200 mmHg (7, 8). Again, in this dog, the rapid improvement of clinical signs was less consistent with ALI or ARDS; therefore, NPE was suspected.

Neurogenic pulmonary edema has a different pathophysiologic basis compared to ALI and ARDS, and in many cases a substantially better prognosis, especially compared to patients with ARDS (27, 28). NPE can occur after injury to the CNS, with clinical signs developing within minutes to hours after injury in the early form, or within 12–24 h with the delayed form (23). Typically, the patient becomes acutely dyspneic, tachypneic, hypoxic within minutes with or without expectoration of pink frothy sputum (23). The prognosis depends on the severity and nature of the underlying neurologic injury, but in many cases NPE resolves rapidly (23, 27, 28). The pathophysiology is not completely understood but increased intracranial pressure from CNS injury results in ischemia and activation of cerebral NPE trigger zones. This can lead to a transient release of massive amounts of catecholamines which results in intense but often momentary systemic vasoconstriction, that shifts blood from the systemic circulation to the low resistance pulmonary microcirculation. This results in local hydrostatic injuries to the pulmonary capillary endothelium and extravasation of protein-rich fluid into the alveoli as well as intra-alveolar hemorrhage (23, 24, 27). The increase in systemic vascular tone may be transitory, and interestingly is often not documented clinically (23, 27). NPE has also been suspected in hunting dogs, with a proposed similar mechanism being release of high concentrations of catecholamines secondary to either excitement, exercise-induced stress, or from seizures secondary to hypoglycemia (29–32). There are also cases of NPE where this transient and often undetected sympathetic overload has been speculated to have injured the left ventricle, leading to a rapidly reversible stress cardiomyopathy called Takotsubo's cardiomyopathy, with development of pulmonary edema (23). Hyperkalemia affecting myocardiocyte function and possibly causing arrhythmias could have also contributed to a hypothetical cardiac dysfunction in this patient. Furthermore, the hypoglycemia and hyponatremia that resulted from hypoadrenocorticism may also have led to CNS impairment and suspected NPE (6). The patient reported on had markedly depressed mentation for the first 24 h without other obvious neurologic signs (such as nystagmus, convulsive seizures), and although the hypoglycemia was only mild at the time of initial presentation, given the state of shock, the hypoglycemia was relatively severe and may well have been more severe prior to presentation. In people, there are reports of hypoglycemia induced NPE secondary to an insulin overdose in diabetics and with the abandoned insulin shock therapy for schizophrenia (24, 33–35). Hypoglycemia can result in cranial ischemia and activation of NPE trigger zones, leading to massive release of catecholamines and shifts blood from the systemic circulation to the low resistance pulmonary microcirculation (29–32). Hypoglycemia may also alter cellular metabolism leading to direct damage to the alveolo-capillary membranes. The reported hyponatremia, exacerbated by the IV fluid therapy in this case, can also lead to encephalopathy and NPE (10, 26). This has been well described in marathon runners who develop exercise-induced hyponatremia from excessive water intake and inappropriate secretion of ADH (36, 37). Microthrombi formation within pulmonary vasculature may further play a role by leading to a substantial increase in the pulmonary hydrostatic pressure. In the reported case, the thrombocytopenia and mild elevations in PT and PTT are supportive of SIRS/DIC and could support this hypothesis.

Main consideration for the cause of the NCPE was given to NPE, but other contributors such as panhypoproteinemia from hypocortisolemia and volume overload were possible. However, more significant hypoalbuminemia is typically required (serum albumin of <10–15 g/L) and cavitary effusions and peripheral edema are expected rather than pulmonary edema. In addition, when NCPE developed, the patient was clinically hypovolemic and hypotensive (MAP < 60 mmHg), there was evidence of hypovolemia on the thoracic radiographs and no other evidence to support volume overload (such as peripheral edema or chemosis). Furthermore, the hypoalbuminemia was not accompanied by a corresponding drop in either the azotemia or hyperkalemia. It is more likely that the hypoproteinemia and hypoalbuminemia occurred as a consequence of exudation of protein-rich fluid into the lungs and gastrointestinal tract, and from the reported melena, rather than from dilution. One study suggested that hypoproteinemia that is reversible during recovery, as seen in this case, is a marker for NCPE and that trending of the serum total protein concentration may be useful for differentiating NCPE from CPE (38).

Treatment for NCPE is largely supportive in nature (oxygen supplementation and in some instances mechanical ventilation, judicious fluid support) with careful hemodynamic stabilization (7). Diuretic therapy is typically not effective for NCPE but has been used to promote bronchodilation (7). Furosemide was not administered in this case to prevent exacerbation of hyponatremia and hypotension. Hypertonic saline IV CRI can be useful for treating NCPE due to hyponatremia, but was avoided here due to concerns of raising the plasma sodium concentration too rapidly as this can theoretically result in central pontine myelinosis (39). If the patient did indeed have NPE then administration of dopamine and phenylephrine may have been ill advised due to their effects on alpha-adrenergic receptors, but their administration was felt to be appropriate given the refractory nature of the hypotension and reluctance to exacerbate hyponatremia and pulmonary edema with more aggressive intravascular volume expansion. Oxygen supplementation along with mineralocorticoid and glucocorticoid supplementations was sufficient for management of NCPE in this case.

Despite this case being classic in most respects for a patient presenting in a primary hypoadrenocorticism crisis, refractory hypotension, and subsequent development of non-cardiogenic pulmonary edema complicated the case management. The proposed cause of the non-cardiogenic pulmonary edema was speculated to be neurogenically mediated in this case.

## Data availability statement

The original contributions presented in the study are included in the article/supplementary material, further inquiries can be directed to the corresponding author.

## Ethics statement

Ethical review and approval were not required for the animal study because the study was a case report. Written informed consent was obtained from the owners for publication.

## Author contributions

MP and ES interpreted the patient data and wrote the manuscript. Both authors have approved this manuscript.

## Conflict of interest

The authors declare that the research was conducted in the absence of any commercial or financial relationships that could be construed as a potential conflict of interest.

## Publisher's note

All claims expressed in this article are solely those of the authors and do not necessarily represent those of their affiliated organizations, or those of the publisher, the editors and the reviewers. Any product that may be evaluated in this article, or claim that may be made by its manufacturer, is not guaranteed or endorsed by the publisher.

## Abbreviations

ABG: arterial blood gas
ACTH: adrenocorticotropic hormone
ALI: acute lung injury
ARDS: acute respiratory distress syndrome
CNS: central nervous system
CPE: cardiogenic pulmonary edema
CRI: continuous rate infusion
CRT: capillary refill time
DIC: disseminated intravascular coagulation
FiO_2_: fraction of inspired oxygen
IM: intramuscular
IV: intravenous
NCPE: non-cardiogenic pulmonary edema
NPE: neurogenic pulmonary edema
PO: per os
RDVM: referring veterinarian
SC: subcutaneous.
